# Position in Sleep

**Published:** 1892-12

**Authors:** 


### Position in Sleep.

The necessity for uninterrupted and refreshing sleep is unquestionable. Sleep that is interrupted by whatsoever cause, loses its refreshing power and the person awakes to duty a loser.

It is unnecessary to repeat here the number of hours of sleep required for the different periods of life, but attention will be called to one point frequently overlooked by physicians when directing patients and others concerning their rest. This is POSITION in sleep. When sleeping, it is necessary for breathing, circulation, and often digestion to go on untrammeled—otherwise sleep is disturbed.

The proper position for securing the easiest discharge of all three of these important functions is probably as follows:

Lie on the right side (unless some physical ailment prohibits) with head slightly drawn down on the pillow, which should be five or six inches high. Flex the limbs slightly, allow no joint to rest upon another. Close the lips and breathe through the nostrils, not the mouth, unless nasal stenosis is present (in which event have removed the hypertrophied tissue). Have lights extinguished or removed to an adjacent room with connecting door open; if an hypnotic has been taken all talking and bustle in the room should cease, in fact everything conducive to sleep should be observed. * * * * *

If free *respiration* alone were the desideratum, the lying on the back would allow more freedom of action to the muscles of respiration than any other position, but circulation and digestion have claims to be considered. So the lying on the right side, with the head slightly drawn down on a pillow five or six inches high, will give freer action to the external inter-costal and the cricoarytenoid muscles, and this position throws the stomach and liver downward and slightly forward, relieving the diaphragm of pressure. The arms should not be folded on the chest nor on the abdomen; for in women the chest needs greater freedom from weight than does the lower half of the trunk. This is possibly a provision of value to the *enceinte* woman. The reverse holds for a man, as he can tolerate for a short time only a weight on the lower portion of the trunk. So the upper extremities will rest most comfortably placing the right by the side of the body, with the left lying on the thigh.

*The circulation.*—The heart is beautifully poised in the thorax, swinging as it does in neither a vertical nor horizontal position when one is erect, but on assuming the recumbent posture on the right side it rests as it were in a hammock, lying almost horizontally, its action becoming less labored, its beats less frequent, its movements comparatively free from friction.

The right lung is probably divided into three lobes, so that in the recumbent position and to the right side the heart may press on the inner surface of the middle lobe without interfering with the action of the lower and upper lobes. In this proper position in sleep the liver and the loaded stomach are not harmfully contiguous to the structures between them and the heart.

Flexing to a slight degree the extremities insures an unobstructed flow of arterial blood. Allowing no joint to rest heavily on another insures an unobstructed return of venous blood, and also obviates injury (by pressure) to the nerves. Nerves thus become obtunded and the sleeper is aroused with "cramping" muscles or with his leg or forearm "asleep."

*The digestion.*—Lying on the right side accomplishes rapid evacuation of the stomach and small intestines of their contents, from the fact, of course, that the pyloric orifice hangs downward and to the right, allowing unobstructed passage of food, as fast as digested, into the small intestines, where, during quiet sleep, absorption may take place and the residue pass through the cecal valve and on to the descending colon.—*Med. Jour.*

